# Hepatic Sarcoidosis: Natural History and Management Implications

**DOI:** 10.3389/fmed.2019.00232

**Published:** 2019-10-30

**Authors:** Mai Sedki, Nicholas Fonseca, Priscila Santiago, Liege Diaz, Monica Garcia-Buitrago, Mehdi Mirsaeidi, Cynthia Levy

**Affiliations:** ^1^Department of Internal Medicine, University of Miami Miller School of Medicine, Miami, FL, United States; ^2^Schiff Center for Liver Diseases, University of Miami Miller School of Medicine, Miami, FL, United States; ^3^Division of Gastroenterology, University of Miami Miller School of Medicine, Miami, FL, United States; ^4^Department of Pathology, University of Miami Miller School of Medicine, Miami, FL, United States; ^5^Division of Pulmonology and Critical Care, University of Miami Miller School of Medicine, Miami, FL, United States; ^6^Division of Hepatology, University of Miami Miller School of Medicine, Miami, FL, United Statesxs

**Keywords:** hepatic sarcoidosis, outcomes, treatment, AMA-negative PBC, hepatic granuloma

## Abstract

**Introduction:** Hepatic granulomas are common in patients with sarcoidosis, but clinically significant liver disease is uncommon and poorly studied. We aimed to characterize the frequency and clinical course of hepatic sarcoidosis in an ethnically diverse population.

**Methods:** This is a retrospective study including all cases of hepatic sarcoidosis in a single center. The median follow-up time was 49 months (4–121). Cases were identified based on ICD-9 and ICD-10 codes for granulomatous hepatitis, sarcoidosis, and hepatic sarcoidosis. The Chi-square and Wilcoxon-signed rank tests were used as indicated to assess for differences between groups.

**Results:** Of 286 patients with sarcoidosis, 27 had hepatic involvement; 78% were female and 48% African American. The most common pattern of liver tests abnormalities was cholestatic. Ten patients had clinically significant hepatic involvement: cirrhosis in seven (25.9%), portal hypertension in nine (33%), and portal vein thrombosis in one (3.7%). Sex, race, and ethnicity were not associated with an increased risk of hepatic involvement or symptomatic hepatic sarcoidosis. Most patients received medical treatment, most commonly oral glucocorticoids. At the end of the follow-up period, all patients were alive but two had undergone liver transplantation due to complications of hepatic sarcoidosis. Three patients with hepatic sarcoidosis had initially been classified as AMA-negative PBC.

**Conclusions:** Hepatic sarcoidosis was found in 9.4% of patients with sarcoidosis and was clinically significant in 37% of those. Identifying and monitoring hepatic sarcoidosis is crucial given its potential complications.

## Introduction

Sarcoidosis is a systemic granulomatous disease of unknown etiology characterized by the presence of non-caseating granulomas in affected organs (1). Although hepatic granulomas can be found in 50–65% of patients with sarcoidosis, the clinical consequences of hepatic involvement are variable and symptomatic disease occurs in only 5–15% of patients (2–4). Given the spectrum of disease manifestation and the invasive nature of confirmatory tests, the diagnosis of hepatic sarcoidosis is often delayed and may be under-reported (4).

Most patients with hepatic sarcoidosis have mild biochemical abnormalities including an elevated alkaline phosphatase (ALP) and gamma glutamyl transpeptidase (GGT) (5–8). Liver biopsy is usually reserved for patients with moderate or severe liver test derangements (8). The non-caseating granulomas of hepatic sarcoidosis are often located along the portal tract, however, several other conditions can cause granulomatous lesions in the liver. Thus, diagnosis of hepatic sarcoidosis is generally based upon the presence of hepatic granulomas, evidence of multi-organ involvement, negative staining and culture for acid-fast bacilli and other bacterial and fungal infections, and exclusion of liver malignancy and drug-induced granulomas (9).

Clinical manifestations of hepatic sarcoidosis vary from asymptomatic disease to severe complications due to cirrhosis and/or portal hypertension. The natural history of hepatic sarcoidosis is poorly understood, with important differences reported across diverse racial and ethnic backgrounds. Hepatic involvement is reportedly more frequent and more severe among African Americans. The aim of this study was to describe the frequency of hepatic sarcoidosis and its clinical course in an ethnically diverse population in a single center in the Southeast of the United States and to identify predictors of prognosis among patients with hepatic sarcoidosis.

## Materials and Methods

### Data Source and Patient Selection

We aimed to identify patients with sarcoidosis and patients with granulomatous hepatitis of all causes. Patients were identified through ICD-9 and ICD-10 codes D86.9 (sarcoidosis, unspecified), D86.8 (sarcoidosis of other sites), and K75.3 (granulomatous hepatitis), seen between January 1st, 2010 and December 1st, 2016 at the University of Miami/Jackson Memorial Hospital. After a manual chart review, patients older than 18 years of age with a diagnosis of sarcoidosis or granulomatous hepatitis were included in the study. In addition, those with granulomatous hepatitis were categorized according to etiology, as follows: autoimmune disorders, systemic infections, malignancies, drug-induced, or idiopathic causes.

Patients diagnosed with sarcoidosis were further evaluated for hepatic involvement. To confirm the diagnosis of hepatic sarcoidosis, we collected data on the hepatic lesions seen in imaging studies, characteristics of the liver granulomas in biopsies, clinical and/or laboratory evidence of liver involvement, and extra-hepatic manifestations of systemic sarcoidosis. For the purpose of this study, hepatic sarcoidosis was defined as “definitive” if biopsy-proven, and “suspected” when the clinical history and objective laboratory data suggested sarcoidosis and no alternative diagnosis existed.

**Figure 1:**
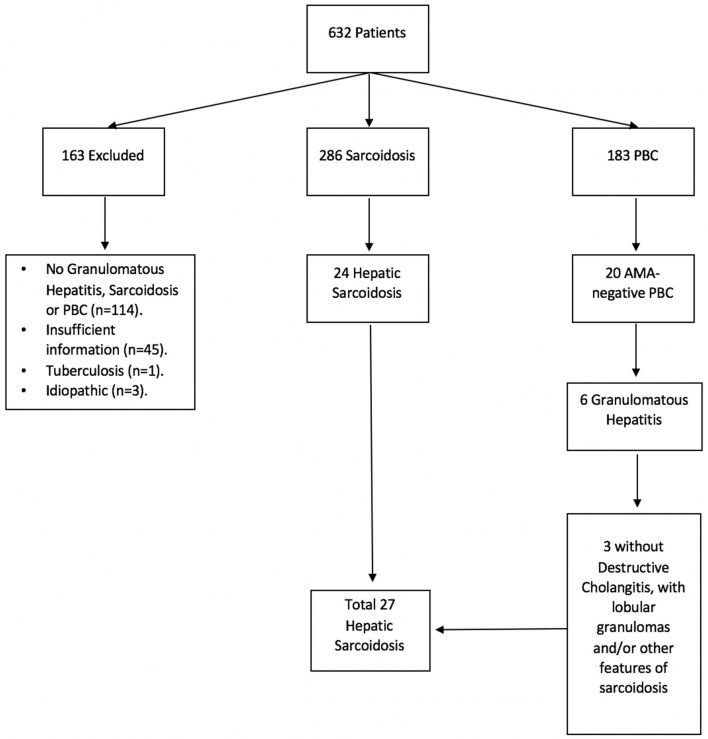
Hepatic sarcoidosis patient selection. A total of 632 medical records were identified under ICD-9 and ICD-10 codes for sarcoidosis, granulomatous hepatitis, and primary biliary cholangitis (PBC). Of these, 286 were found to have sarcoidosis and 183 had PBC, of whom 20 tested negative for anti-mitochondrial antibody (AMA). Six patients with AMA-negative PBC had granulomatous changes on histology, of whom 3 failed to demonstrate destructive cholangitis and therefore were diagnosed with sarcoidosis instead.

Due to the clinical and histological similarities between primary biliary cholangitis (PBC) and hepatic sarcoidosis, we also retrieved a list of patients with ICD-9 code K74.3 (PBC) in an effort to identify cases of hepatic sarcoidosis potentially misdiagnosed as anti-mitochondrial antibody (AMA)-negative PBC (Figure 1).

### Data Collection and Statistical Analysis

Data on demographic characteristics, comorbid conditions, extrahepatic manifestations of hepatic sarcoidosis, liver biochemical tests at baseline and at last follow-up, treatment, and clinical outcomes were collected. For patients with AMA-negative PBC, additional information on associated co-morbidities (sicca syndrome, scleroderma, Raynaud's disease, thyroid disease, rheumatoid arthritis and celiac disease) and bile duct abnormalities on histology were also collected.

Descriptive statistics were used to summarize the cohort demographics. When appropriate, categorical variables were analyzed using chi-square or Fisher's exact test, and continuous variables using Student *t*-test or Wilcoxon signed rank test. A two-sided *p*-value < 0.05 was considered statistically significant. Analyses were performed using IBM SPSS Statistics V25.0 (Armonk, NY).

## Results

A total of 632 medical records were identified as having a diagnosis of sarcoidosis and/or granulomatous hepatitis by ICD code. Of those, 286 had confirmed sarcoidosis, 163 AMA-positive PBC, 20 AMA-negative PBC, 3 idiopathic granulomas, 1 tuberculosis, and 159 had insufficient information or incorrect diagnosis, with lack of granulomas, sarcoidosis or PBC (Figure 1). Patients with AMA-positive PBC, idiopathic granulomatous hepatitis, tuberculosis and those with insufficient/incorrect diagnosis were excluded from further analyses (*n* = 326).

Of the 286 patients with sarcoidosis, 198 (69%) were female, 145 (51%) Caucasian, 122 (43%) African Americans, and 63 (22%) Hispanics. Only 27 had hepatic involvement: 18 with a definitive diagnosis based on biopsy findings and nine with a suspected diagnosis: six were diagnosed based on medical history and three were initially misdiagnosed with AMA-negative PBC which was later modified to hepatic sarcoidosis. Median time from sarcoidosis diagnosis to determination of hepatic involvement was 5.7 months (range: 0.0, 158.3 months). Median duration of follow-up from diagnosis of hepatic sarcoidosis to last visit was 4.0 years (range: 1.2, 8.9 years).

### Baseline Characteristics of Patients With Hepatic Sarcoidosis

In our cohort of 27 patients, 20 were female (74%), 13 were African American (48%), 10 were Caucasian (37%) and 4 (15%) were Hispanics. The median age at diagnosis of hepatic sarcoidosis was 45.7 years (range: 23.9–72.5 years). Laboratory tests at baseline and follow-up are illustrated in Table 1. Pulmonary involvement was the most commonly reported manifestation of systemic sarcoidosis in our cohort of patients diagnosed with hepatic sarcoid, followed by lymphatic, ocular, cardiac, renal, and cutaneous involvement (Table 2). Inflammatory bowel disease was present in only one patient, whereas four patients had pancreatitis. Frequent comorbidities included hypertension and diabetes mellitus.

**Table 1:** Pertinent laboratory tests.

**Laboratory test**	**Baseline**	**Last follow-up**
ALP	528 ± 481 IU/L	204 ± 157 IU/L
ALT	60 ± 38 IU/L	28 ± 16 IU/L
AST	54 ± 27 IU/L	37 ± 23 IU/L
Albumin	3.72 ± 0.7 g/dL	4.0 ± 0.4 g/dL
Bilirubin	1.34 ± 1.9 mg/dL	1.08 ± 1.1 mg/dL
Creatinine	2.22 ± 3.2 mg/dL	1.68 ± 2.0 mg/dL
INR	1.04 ± 0.07	1.09 ± 0.12
Platelets	213 ± 98 × 10^3^/μL	172 ± 62 × 10^3^/μL
ACE	100 ± 82 U/L	79 ± 62 U/L

*ALP, alkaline phosphatase; ALT, alanine aminotransferase; AST, aspartate aminotransferase; INR, international normalized ratio; ACE, angiotensin converting enzyme. Results are provided as median ± standard deviation*.

**Table 2:** Organ involvement by sarcoidosis.

**Extra-hepatic involvement**	***N* (%)**
Pulmonary	15 (56%)
Lymphatic	11 (41%)
Ocular	7 (26%)
Cardiac	3 (11%)
Renal	2 (7%)
Cutaneous	(4%)

The most common laboratory abnormality was an elevation in serum ALP, or a cholestatic pattern of liver enzyme abnormalities. Median serum ALP at baseline was 3.5 times the upper limit of normal, associated with minimal elevation in transaminases (Table 2). Anti-nuclear antibody (ANA) was tested in 13 patients and was present in 6 (46%). Angiotensin converting enzyme (ACE) level was elevated in nine of the 12 patients whose test was available. Although liver biopsy was performed in every patient, biopsy data was only available for 18 patients; 100% of the biopsies revealed non-caseating granulomas mostly located in a portal or multifocal distribution and none showed lipogranulomas or fibrin rings. Ductopenia was evident in four patients, two of whom also had bile duct injury and one with florid duct lesions.

At presentation, the most frequently reported symptom was fatigue, followed by pruritus, weight loss, and hepatomegaly equally (Table 3). Ten patients had clinically significant hepatic involvement: cirrhosis was diagnosed in seven (26%), portal hypertension in nine (33%), and one patient had portal vein thrombosis (4%).

**Table 3:** Clinical findings.

**Clinical presentation**	***N* (%) at baseline**	***N* (%) at follow-up**
Fatigue	12 (44%)	11 (41%)
Pruritus	7 (26%)	2 (7%)
Weight loss	7 (26%)	3 (11%)
Hepatomegaly	7 (26%)	6 (22%)
Dyspnea	6 (22%)	4 (15%)
Cough	6 (22%)	5 (19%)
Abdominal pain	5 (19%)	5 (19%)
Night sweats	4 (15%)	1 (4%)
Fever	4 (15%)	0
Esophageal varices	3 (11%)	8 (30%)
Ascites	2 (7%)	1 (4%)
Jaundice	1 (4%)	3 (11%)
Chest pain	1 (4%)	1 (4%)
Encephalopathy	0	1 (4%)

### Outcomes

Table 1 shows median values for aspartate aminotransferase (AST), alanine aminotransferase (ALT), ALP and total bilirubin (TB) at baseline and at last follow-up. Of the 22 patients who had an elevated ALP at diagnosis, 11 normalized by the end of our study period whereas nine had lower ALP but not within the normal range. Of those 11 patients, eight patients were observed to have a corresponding reduction in serum AST and ALT levels with an average decrease of 32 and 48 U/L, respectively. At the end of the follow-up period, 49 months (4–121), 67% and 83% of patients had normal AST and ALT, respectively, compared to 35% and 17% at diagnosis. Hyperbilirubinemia was observed in three patients at diagnosis and in two at the end of follow-up. No prolongation of prothrombin time was observed. Thrombocytopenia was reported in four patients at diagnosis, three of whom were found to have splenomegaly. At the end of follow-up, seven patients had thrombocytopenia of whom five had splenomegaly. One patient had splenomegaly without evidence of thrombocytopenia. Five additional patients were found to have esophageal varices by the end of the follow-up period. On the other hand, a smaller proportion of patients reported pruritus and weight loss at the end of follow up when compared to symptoms at diagnosis.

### Effect of Treatment

A total of 21 patients were medically treated with one or more agents during our follow-up period; six patients were never treated medically. Oral glucocorticoids were the most frequently used in 17 patients (63%), followed by antimetabolites in 15 patients (56%), and biologic agents in five patients (19%). Median change in ALP from baseline to last follow-up for patients treated with anti-metabolites, steroids, and biologics was −163.5, −219, and −108, respectively, compared to −162.5 in untreated patients (*p* = 0.29, 1.0, and 0.62 compared to no treatment 0.29). Antimetabolites elicited a statistically significant change in ALP in individuals with preexisting ALP elevation (*p* < 0.05). On the other hand, patients receiving oral glucocorticoids or biologic agents showed some lowering in their ALP level which was not statistically significant, *p* = 0.09, 0.11, respectively.

At the end of the follow-up period, all patients were alive and two patients received orthotopic liver transplant (OTL) due to complications of hepatic sarcoidosis. The first is a 56-year-old African American woman with well-established cutaneous and pulmonary sarcoidosis, hypertension, hyperlipidemia, depression, and Parkinson's disorder, who initially presented with complications of cirrhosis including esophageal varices, encephalopathy, and hepatopulmonary syndrome. Although the treating physician was concerned about AMA-negative PBC, her explant showed cirrhosis with moderate chronic inflammation, ductopenia, intracellular cholestasis, with multiple portal and lobular non-necrotizing granulomas. There was no evidence of destructive cholangitis. Non-necrotizing granulomas involving the fibroadipose tissue surrounding hilar vessels were noted. Her post-operative course was complicated by delayed graft function, two embolic strokes, and wound infection, but she eventually recovered.

The second patient is a 65-year-old Caucasian male with a long-standing 20-year history of extensive granulomatous liver disease which eventually led to end-stage liver disease. He had been treated with ursodiol, multiple courses of steroids and methotrexate without success; he underwent OLT due to complications of portal hypertension. His explant showed a cirrhotic liver with marked cholestasis, ductopenia, focal periductal fibrosis, mild chronic inflammatory infiltrates, and non-caseating granulomatous inflammation. The post-operative course was remarkable for partial wound dehiscence, an episode of cholangitis, and acute cellular rejection.

### Does Race or Ethnicity Predict Hepatic Involvement or Disease Progression in Sarcoidosis?

Sex, race, and ethnicity were not associated with an increased risk of hepatic involvement in patients with systemic sarcoidosis (*p* = 0.66, 0.79, 0.15, respectively). In our cohort there was no statistically significant association between sex (*p* = 0.49) or race (*p* = 0.86) and clinically significant hepatic sarcoidosis.

### Discernment From AMA-Negative Primary Biliary Cholangitis

Of the 20 cases of AMA-negative PBC, six had granulomatous hepatitis on liver biopsy and three of those did not have clear evidence of destructive cholangitis. Rather, the biopsy revealed non-caseating granulomas involving both the portal space and hepatic lobule. Upon further review, those three patients were deemed to have hepatic sarcoidosis due to evidence of systemic organ involvement with sarcoidosis and absence of destructive cholangitis on liver biopsy.

**Figure 2:**
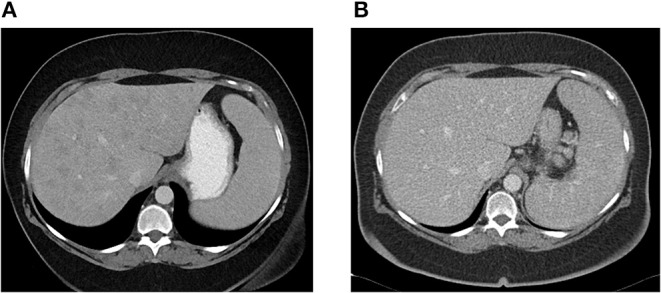
**(A)** Abdominal CT showing innumerable low attenuation nodules consistent with hepatic sarcoidosis. Patient was previously diagnosed with AMA-negative PBC, later re-evaluated and diagnosed as sarcoidosis. **(B)** Abdominal CT 6 months later, after patient was successfully treated with azathioprine with resolution of hepatic nodules.

**Figure 3:**
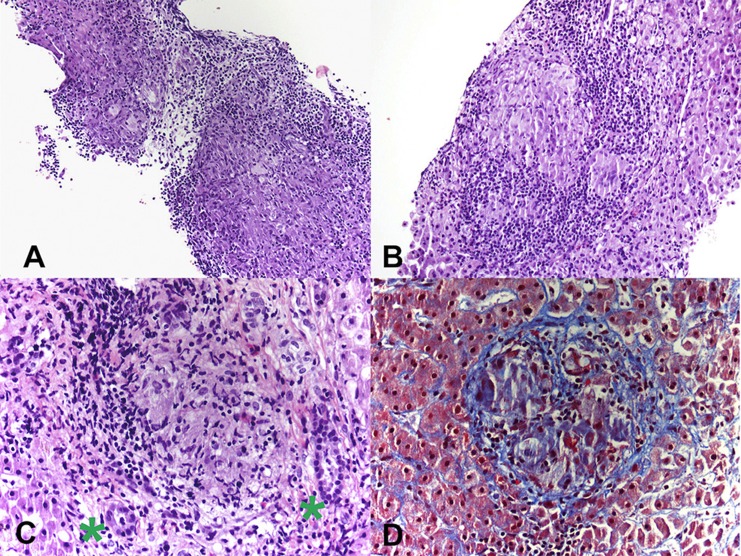
**(A)** Photomicrograph from liver biopsy showing multiple coalescing non-necrotizing granulomas (Hematoxylin & Eosin, 10×). **(B)** Lobular non-necrotizing granuloma with tightly packed epithelioid cells surrounded by lymphocytes (Hematoxylin & Eosin, 20×). **(C)** Portal non-necrotizing granuloma adjacent to uninvolved bile ducts (green stars) (Hematoxylin & Eosin, 40×). **(D)** Mild peri-granulomatous fibrosis (Trichrome stain, 40×).

Patient CE was the most striking example: a 71-year-old female who presented 10 years prior with elevated ALP and liver biopsy showing portal granulomas, some bile duct loss and portal fibrosis. She was diagnosed with AMA-negative PBC, initially with complete response to ursodeoxycholic acid. Five years later, due to persistent elevation of ALP, additional work-up was performed with repeat liver biopsy, ACE level and CT scan (Figure 2). The biopsy showed multiple coalescing well-formed epithelioid granulomas in all zones. Marked lymphocytic infiltrates were present in portal tracts and in the lobules but florid duct lesions were not seen (Figure 3). The ACE level was elevated, 1.6–2 times the upper limit of normal, and the CT abdomen showed innumerable low-attenuation nodules of varying sizes scattered through the right and left lobes of the liver, worsening lymphadenopathy in the upper abdomen and mild splenomegaly (Figure 2). Those changes were definitely not present on prior imaging.

The second patient, MD, an African American female who was 32 years-old when diagnosed with AMA-negative PBC based on chronic cholestasis and liver biopsy with granulomatous hepatitis. She was treated with ursodeoxycholic acid, with incomplete biochemical response. Upon review of liver biopsy, she did not have destructive cholangitis and the granulomas were predominantly lobular.

Finally, the third patient was the 56-year-old female transplant recipient described above.

## Discussion

In the present study, clinically significant hepatic sarcoidosis was found in 9.4% of an ethnically diverse group of patients with sarcoidosis. A significant proportion of patients with hepatic sarcoidosis lacked lung involvement. Sex, race, and ethnicity were not associated with an increased risk of hepatic involvement or symptomatic hepatic sarcoidosis. Liver test abnormalities predominantly involved a cholestatic pattern. A quarter of our patients with hepatic sarcoidosis already had cirrhosis at the time of diagnosis. Most patients received medical treatment, most commonly oral glucocorticoids, with 83% responding well to treatment evidenced by normalization in liver biochemistries.

In a recent study by Ungprasert et al., hepatic involvement in sarcoidosis was seen in 6% of the patients, with the majority being asymptomatic and only three patients presented with isolated hepatic sarcoidosis without intra-thoracic disease (10). Kennedy et al. reported that half of their cohort of 180 patients with sarcoidosis had abnormal liver biochemical tests attributable to hepatic sarcoid and 13% had liver involvement without lung disease (5). Comparatively, 22.2% of our patients had liver involvement without lung disease.

Despite reports of African Americans having up to a 3-fold increased risk of developing sarcoidosis compared to Caucasians (2, 11) and an association between African American race and severity of liver involvement (12), we found no significant impact of race on the risk of developing hepatic sarcoidosis nor on its severity. Although our study is limited by a relatively small sample size, almost half of patients with hepatic sarcoidosis were African Americans and yet we did not find a difference in the proportion of patients with cirrhosis and/or portal hypertension based on race or ethnicity.

Routine laboratory evaluation in sarcoidosis is often non-specific. Patients with hepatic sarcoidosis have abnormal liver biochemistries, with ALP being most commonly affected (13, 14). Fetzer et al. reported ALP elevation in 50% of their cohort of 39 patients with hepatic sarcoidosis (4). In fact, up to 95% of patients with hepatic sarcoidosis have elevations in their ALP up to three-times the upper limit of normal (8, 10, 15). Similarly, we found that cholestasis was the most common laboratory abnormality in our population. The degree of liver test abnormality appears to be related to the degree of granulomatous inflammation and the degree of fibrosis (8). The role of ACE levels in diagnosing or managing sarcoidosis is controversial, as ACE level is not a sensitive enough test for the diagnosis of systemic sarcoidosis (10, 16–18). However, increased levels may be useful in supporting a suspected diagnosis. Fetzer et al. reported ACE level elevation in 65.5% of 39 patients with hepatic sarcoidosis (4). In our study, ACE level was available for only 12 patients and elevated in nine (75%).

Clinical manifestations of hepatic sarcoidosis include non-specific symptoms, such as fatigue, malaise, weight loss, abdominal pain and pruritus. Additionally, patients may also present with hepatomegaly and jaundice. The most frequently reported symptom in our cohort was fatigue, present in 44% of the patients, followed by pruritus, weight loss, dyspnea, and cough. While Ungprasert et al. reported that none of the patients experienced cholestatic symptoms despite the majority having elevated ALP (10), at least a quarter of the patients in our cohort experienced pruritus and three patients had jaundice. Although the course of the disease tends to be benign for most patients, some may develop cholestatic liver disease, cirrhosis, Budd-Chiari syndrome and/or hepatic vein thrombosis, eventually requiring liver transplantation (1, 6, 7, 19). Furthermore, portal hypertension can develop due to biliary cirrhosis or compression of the portal vein by involved portal lymph nodes or parenchymal granulomas (19, 20).

A quarter of our patients with hepatic sarcoidosis already had cirrhosis at the time of diagnosis, and a third had portal hypertension with esophageal varices during the clinical course of the disease; a subset of patients presented with portal hypertension in the absence of cirrhosis. Cirrhosis due to sarcoid-related portal hypertension can vary from 27 to 100% (5, 10). In another study including 35 patients with hepatic sarcoidosis, half had portal hypertension without evidence of cirrhosis (21). In this setting, non-cirrhotic portal hypertension occurs as pre-sinusoidal granulomas cause mass effect and restriction in portal flow through the sinusoids (pre-sinusoidal portal hypertension).

The treatment of hepatic sarcoidosis is challenging. Without randomized controlled trials it has been difficult to draw conclusions on the efficacy and long-term benefits of glucocorticoids and/or other immunosuppressants. Glucocorticoids are often the initial drug of choice in the treatment of most forms of extra-thoracic sarcoidosis, an initial dose of 20–40 mg of daily prednisone or an equivalent is usually adequate (22). Nevertheless, the response to conventional immunosuppression is variable and unpredictable. Kennedy et al. found that 49% were treated with oral glucocorticoids and 62% had a complete biochemical response. In our study 17 (63%) patients received oral glucocorticoids, 15 (56%) anti-metabolites, 5 (19%) biologic agents (anti-TNF-alpha therapy), and 3 (11%) received a combination of all three therapeutic agents. Interestingly, 83% responded well to treatment with normalization of liver biochemistries. The clear endpoint of treatment is to control symptoms and ultimately prevent progression to cirrhosis and/or portal hypertension. In that regard, fewer patients were symptomatic at the end of follow-up period and 83% of treated patients normalized liver biochemistries. However, it would be speculative to discuss the effect of therapy in this small subset of patients with hepatic sarcoidosis, a quarter of whom already presented with cirrhosis at baseline due to delay in diagnosis.

**Figure 4:**
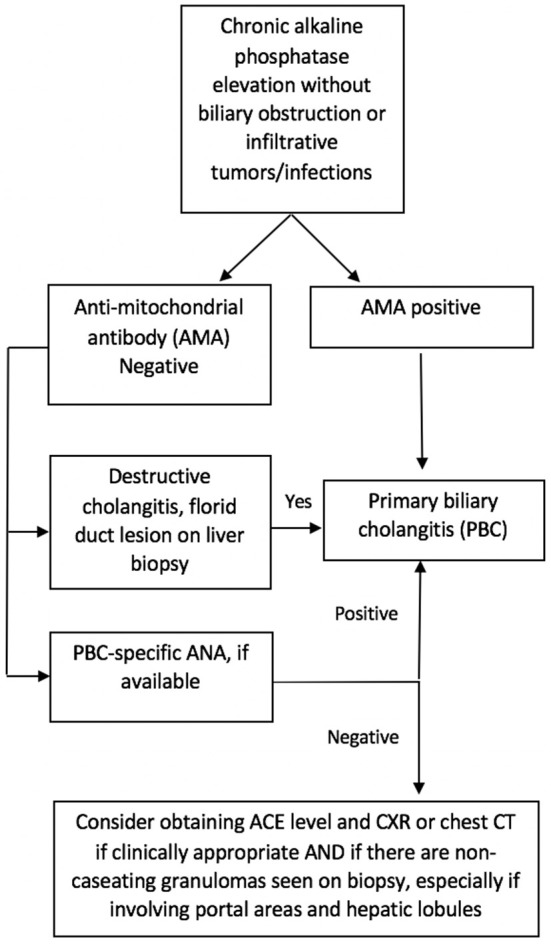
Suggested algorithm for differentiation of AMA-negative primary biliary cholangitis (PBC) and hepatic sarcoidosis.

Since sarcoidosis often presents with an elevation of cholestatic liver enzymes, it may resemble PBC and even primary sclerosing cholangitis (PSC); in addition, both can coexist with sarcoidosis (14, 21). In contrast with sarcoidosis and PBC, the classic form of PSC is characterized by large bile duct involvement, making the diagnosis easier to distinguish. However, sarcoidosis can mimic PBC, leading to diagnostic dilemmas in AMA-negative patients. In our study, we identified three patients who were initially diagnosed with AMA-negative PBC but did not meet the American Association for the Study of Liver Diseases (AASLD) criteria for the diagnosis of PBC (23). The most challenging cases are middle-aged Caucasian females presenting with pruritus and elevated ALP, since this is the typical age and sex distribution of PBC. Our study suggests that further consideration and investigation for sarcoidosis is indicated in patients with intra-hepatic cholestasis, negative AMA test, and evidence of granulomas on liver biopsy, especially if these are predominantly lobular. Pulmonary imaging and measurement of serum ACE level may suffice as an initial evaluation. Furthermore, if ANA is present in these patients, further testing for PBC-specific ANAs (gp210 and sp100) is recommended. An algorithm is proposed to aid clinicians in this diagnostic process (Figure 4).

In conclusion, our study highlights the importance of correctly identifying hepatic sarcoidosis, given increased morbidity when associated with cirrhosis and/or portal hypertension. Race, sex, and ethnicity may not predict hepatic involvement and disease progression. When appropriate, special attention should be given to AMA-negative PBC patients to exclude the possibility of hepatic sarcoidosis.

## Data Availability Statement

The raw data supporting the conclusions of this manuscript will be made available by the authors, without undue reservation, to any qualified researcher.

## Ethics Statement

The studies involving human participants were reviewed and approved by University of Miami Institutional Review Board. Written informed consent for participation was not required for this study in accordance with the national legislation and the institutional requirements.

## Author Contributions

CL designed the study concept. Data collection was performed by NF, PS, and LD. Analysis and interpretation of data was performed by MS and CL. Draft of the manuscript was performed by NF and MS. MG-B, MM, and CL provided the critical revision of the manuscript.

### Conflict of Interest

The authors declare that the research was conducted in the absence of any commercial or financial relationships that could be construed as a potential conflict of interest.
